# Which preoperative screening tool should be applied to older patients undergoing elective surgery to predict short-term postoperative outcomes? Lessons from systematic reviews, meta-analyses and guidelines

**DOI:** 10.1007/s11739-020-02415-y

**Published:** 2020-07-01

**Authors:** Rachel Aitken, Nur-Shirin Harun, Andrea Britta Maier

**Affiliations:** 1 Department of Medicine and Aged Care, The University of Melbourne, The Royal Melbourne Hospital, @AgeMelbourne, Parkville, VIC Australia; 2 Department of Medicine, The Royal Melbourne Hospital, Melbourne, Australia; 3 Department of Human Movement Sciences, Faculty of Behavioural and Movement Sciences, Vrije Universiteit Amsterdam, Amsterdam Movement Sciences, @AgeAmsterdam, Amsterdam, The Netherlands

**Keywords:** Geriatric assessment, Aged, Screening tool, Preoperative care, Surgery, Frailty

## Abstract

**Background:**

Older surgical patients have a higher risk of postoperative mortality and morbidity compared to younger patients. Timely identification of high-risk patients facilitates comprehensive preoperative evaluation, optimization, and resource allocation to help reduce this risk. This review aims to identify a preoperative screening tool for older patients undergoing elective surgery predictive of poor short-term postoperative outcomes.

**Methods:**

A scoping review was conducted. An Ovid MEDLINE search was used to identify systematic reviews or meta-analyses comprising older elective patients in at least two different surgical settings. International guidelines were reviewed for recommendations regarding preoperative tools in this population.

**Results:**

Over 50 screening tools were identified. The majority showed a positive association with short-term postoperative mortality and morbidity in older patients. The most commonly described tools were the American Society of Anesthesiologists Physical Status (ASA-PS), frailty tools and domain-specific tools administered as part of comprehensive geriatric assessment (CGA). Due to heterogeneity in outcome measures and statistical methodology the predictive capacity between tools could not be compared. International guidelines described a comprehensive preoperative approach incorporating domain-specific tools rather than recommending a screening tool.

**Conclusion:**

Multiple tools were associated with poor short-term postoperative outcomes in older elective surgical patients. No single superior tool could be identified. Frailty, cognitive and/or functional tools were most frequently utilized.

**Electronic supplementary material:**

The online version of this article (10.1007/s11739-020-02415-y) contains supplementary material, which is available to authorized users.

## Background

Older people constitute the most rapidly growing group throughout the developed world [1]. This evolving demographic shift has led to an increased demand for surgery in older patients [2]. Older patients are more likely to suffer from multi-morbidity, frailty, cognitive and functional impairment [3]. As a result, they have poorer postoperative outcomes including higher mortality and complication rates, a prolonged length of stay and increased likelihood of discharge to supported accommodation compared to younger patients [4, 5]. Equally important measures of recovery, such as health-related quality of life, are infrequently reported and poorly defined [6].

Efficient and effective screening of older patients who may be at increased risk of these poor postoperative outcomes is a current challenge facing clinicians and service providers. Identification of high-risk older patients aims to improve postoperative outcomes through targeted comprehensive geriatric assessment (CGA) and medical optimization, shared decision-making, engagement of the perioperative multidisciplinary team and allocation of critical care resources [7–9]. Conversely, not all older patients will benefit from these interventions.

There is lack of consensus on which screening tools should be applied to older patients in an elective surgical setting [10]. Although there is an abundance of tools in existence, many are narrowly targeted towards specific surgical subtypes or require specialist training to administer. Thus, a preoperative assessment tool that can be easily and broadly applied to older elective surgical patients with a high ability to predict poor postoperative outcomes is sought.

This scoping review aims to examine the ability of preoperative assessment tools to predict poor short-term postoperative outcomes in older patients undergoing elective surgery and to determine if a single best screening tool can be recommended in this cohort. We also aim to summarize recommendations for the use of these preoperative assessment tools in relevant international guidelines on the perioperative care of the older patient.

## Methods

Given the broad research question with anticipated heterogenous results, a scoping review based on Arksey and O’Malley’s framework was conducted [11].

### Search strategy

We searched Ovid MEDLINE for systematic reviews and meta-analyses of preoperative tools applied to older patients undergoing elective surgery published between January 2000 and 8 February 2019. The literature search was conducted with assistance from a health sciences librarian. Keywords were combined with MeSH search terms ‘surgical procedures, operative’, ‘elective surgical procedures’, ‘risk assessment or risk factors’, ‘outcome assessment (health care)’, ‘decision support techniques’, ‘postoperative complications’, ‘mortality’, ‘morbidity’, ‘length of stay’ and ‘treatment outcome’. The detailed search string is listed in electronic supplementary material. The inclusion of international guidelines was deemed necessary after the literature search of systematic reviews and meta-analyses lacked a clear consensus on which screening tools were best to use in the population of interest.

### Review procedure

Two investigators (RA, NSH) screened the titles and abstracts and selected articles for full-text review. Full-text articles were then examined for eligibility. A third researcher (ABM) resolved any differences that could not be decided by consensus. A manual search of the references of eligible articles was also performed. In addition, relevant international guidelines evaluating older patients undergoing elective surgery were screened for recommendations regarding evidence-based preoperative tools.

### Inclusion criteria

Eligible articles consisted of systematic reviews or meta-analyses in which the majority of study participants were older patients undergoing elective surgery. Older patients were defined as a population mean or median age of 60 years or older. If the age range was not stated in the review article, original articles were examined. Screening tools needed to be tested in at least two different elective surgical populations. This ensured the tools were not limited to a specific surgical group and were therefore more broadly applicable. Tools needed to be able to be completed preoperatively. Outcomes of interest were short-term mortality (inpatient mortality, 30-day or 90-day mortality), length of stay and measures of short-term postoperative morbidity such as postoperative complications, postoperative delirium, quality of life and discharge to a care facility.

## Results

**Fig. 1:**
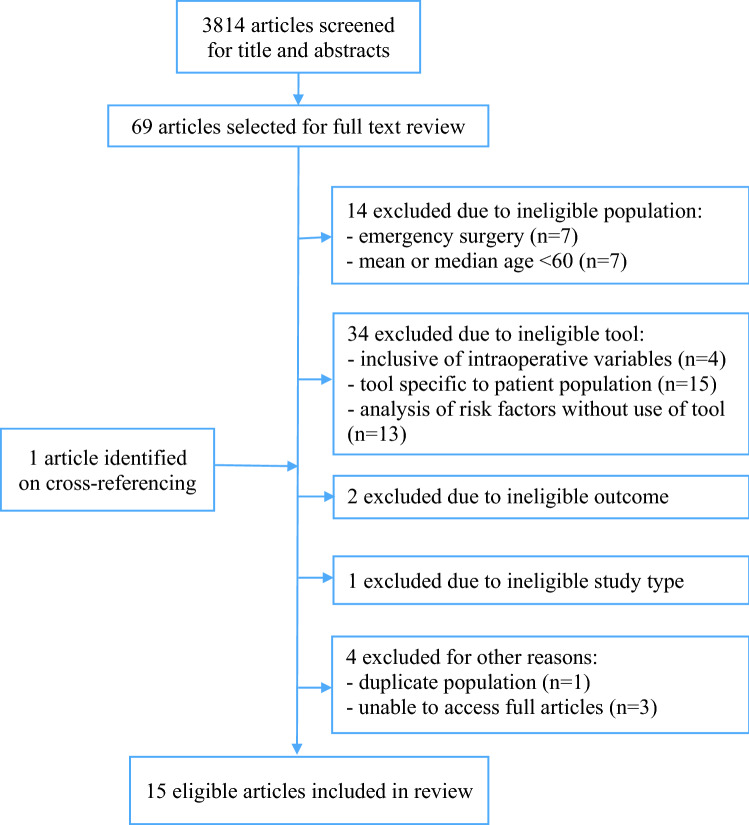
Flowchart of selection of articles for inclusion in review

The literature search yielded 3814 articles. Screening of titles and abstracts resulted in 69 articles selected for full-text review. Following the exclusion of articles based on study type, patient population, tools and outcomes, 15 articles were selected for inclusion [12–26] as detailed in Fig. 1.

More than 50 different preoperative tools were identified. The American Society of Anesthesiologists Physical Status (ASA-PS) tool, several frailty tools and domain-specific tools included as part of CGA were most frequently reported. Characteristics of the included studies are listed in Table 1. Tools and association with postoperative mortality and morbidity are detailed in Table 2.

**Table 1:** Characteristics of systematic reviews and meta-analyses

Author, year	Study design	Surgical Population	Urgency	Age, years	Articles, *N*	Patients, *N*	Tool	Outcome
Abdullahi 2017 [12]	SR	Cardiac	Majority elective	Mean > 65	6	4819	Gait speed, Katz IADL, Mini-Cog, CCI, Anemia, Geriatric syndrome of falls, CAF, FORECAST, TUGT, gait speed, Nagi scale	Mortality (inpatient, 30-day and 1 year), postoperative complications, length of stay
Buignes 2015 [13]	SR	Mixed major	Majority elective	Majority mean ≥ 60	32	NA	mFI, Fried criteria, gait speed, CHS, MSSA4, CAF, Katz IADL, EFS, CGA	Postoperative mortality, postoperative complications, Length of stay
Fagard 2016 [14]	SR	Colorectal cancer	Majority elective	Mean > 65	5	486	Fried, GFI, CGA (Barthel index, NEADL, CIRS, polypharmacy, MNA, MMSE, GDS), Katz, TUGT, CCI, Anemia, Mini-Cog, Albumin, falls	Mortality (30-day, 1 year, 5 year), postoperative complications, length of stay, 30-day readmission
Hewitt 2018 [15]	MA & SR	Gastro-intestinal	Majorityelective	Mean age ≥ 60	9	2281	Physical Frailty Phenotype, DAI, GFI, 7-point clinical frailty score	30-day mortality, postoperative complications, length of stay
Huisman 2017 [16]	SR	Oncology	Elective	Mean ≥ 60	9	NA	CGA domains: function (ADL impairment, ADL, IADL, Barthel, functional limitations, NEADL, falls, TUGT), nutrition (MNA, weight loss, MMC), cognition (MMSE, mini-cog), social support (MOS-SSS), mood (GDS, HADS, MHI), comorbidity (CIRS, SIC, CCI), polypharmacy (> 5), frailty (CGA-based, GFI, frailty phenotype)	Mortality (short-term, long-term, disease-free survival), postoperative complications
Lin 2016 [17]	SR	Mixed major	Majority elective	Mean > 75	23	17,117	Fried, modified CHS, MSSA4, gait speed, Katz, SHERPA-risk, MMSE, MNA, TUGT, BADL, IADL, Comprehensive assessment of frailty, CAF, FORECAST, Balducci, frailty criteria, John Hopkins, CFS, Vulnerable elderly survey, MFS, Addenbrooke’s vascular frailty score, EFS, FI, mFI, KCCQ-OS QoL time	Mortality (in-hospital, 30 day and long term), postoperative complications, length of stay, discharge to institutional care, functional decline, QoL
Oldroyd 2017 [18]	MA & SR	Vascular	Majority Elective	> 65	16	3617	Risk factors including ASA > 2	Postoperative delirium
Panayi 2018 [19]	MA & SR	Mixed major	Majority elective	≥ 60	16	683,487	mFI	30-day mortality, postoperative complications, readmission, discharged to facility
Partridge 2014 [20]	SR	Mixed major	Elective	Majority > 60	5	1364	CGA tools: MMSE, Barthel, ADL, IADL, TUGT, MNA, clock, GDS, social support scale	Postoperative complications, length of stay, change in QoL
Sandini 2017 [21]	MA & SR	Mixed major	Elective	Mean age ≥ 65	35	1,153,684	Frailty tools: tools not published in paper. Domains listed: activity, sarcopenia, comorbidities, nutrition, cognition, depression, walking incl	Mortality (90-day, 1 year), 30 day major morbidity
Scholz 2016 [22]	MA & SR	Gastro-intestinal	Elective, mixed	> 65	11	1427	Risk factors including ASA > / = 3, CCI	Postoperative delirium
Sepehri 2014 [23]	SR	Cardiac	Majority elective	> 60	6	4756	Fried, Katz IADL, CAF, mFI, CHS, MSSA4, gait speed, MMGA, MGBE (MMSE, MNA, TUGT, BADL) frailty tools	Mortality (in-hospital, all-cause, 1 year), MACCE, discharge to institution, functional decline
Visser 2015 [24]	SR	Mixed major	Majority elective	Majority mean ≥ 60	30	UNK	Risk factors including ASA grade	Postoperative mortality, postoperative complications
Warnell 2015 [25]	SR	Oesophagectomy	Elective	Majority mean > 60	20	13,887	ASA, POSSUM, P-POSSUM, O-POSSUM, CCI, Karnofsky index	Mortality (in-hospital or 30 day)
Zhu 2017 [26]	MA	Head and neck cancer	Elective	Majority mean > 60	8	1940 (incl controls)	Risk factors including ASA ≥ 3	Postoperative delirium

*IADL* instrumental activities of daily living, *CCI* Charlson comorbidity index, *CAF* Comprehensive Assessment of Frailty, *FORECAST* Frailty predicts death One year after Elective Cardiac Surgery Test, *TUGT* timed up and go test, *mFI* modified frailty index, *CHS* cardiovascular health study frailty tool, *MSSA4* 4 item frailty scale (gait speed, handgrip strength, inactivity, cognitive impairment), *EFS* Edmonton frailty scale, *GFI* Groningen frailty indicator, *NEADL* Nottingham extended ADL scale, *CIRS* cumulative illness rating scale, *SIC* Seattle index of comorbidity, *MNA* mini-nutritional assessment, *MMSE* mini-mental status examination, *DAI* deficit accumulation index, *MMC* mid arm muscle circumference, *MOS-SSS* medical outcomes study social support survey, *GDS* geriatric depression scale, *HADS* hospital anxiety and depression scale, *MHI* mental health inventory, *MFS* Morse fall scale, *MMGA* modified multidimensional geriatric assessment, *MGBE* modified geriatric baseline examination

**Table 2:** Predictive capacity of preoperative assessment tools

Tool	Mortality	Morbidity and length of stay
ASA	AUROC 0.64 [25] OR 1.54–11.6 [24]	*Postop complications:* OR 1.77–7.1 [24] ASA > 2: OR 3.44 (2.02–5.87) [18] ASA-3: Clavien-Dindo 4 OR 6.8 [13] ASA ≥ 3: pooled OR 2.71 (1.64–4.48) [22] *Delirium:* ASA ≥ 3: OR 5.65 (1.57–20.36) [26] *Cardiac arrest:* ASA-3: OR 1.2, ASA-4: OR 3.5, ASA-5: OR 7.5 [13] *Perioperative MI:* ASA-3: OR 3, ASA-4: 6.9, ASA-5: 14.9 [13]
CAF	≤ 11 30d mortality OR 1.1 (1.06–1.2) [12]	
CCI	AUROC 0.57 [25] All-cause mortality HR 1.03 (0.9–1.17) [16]	*Postop complications:* OR 0.93 (− 1.68–3.54) [22]
CGA assessment of frailty	2 frailty markers: 6 mo mortality HR 3.86 (0.41–36.02)–8.88 (1.09–72.29) [16] ≥ 3 markers: 6 mo mortality HR 4.51 (0.49–41.25)–8.5 (1.1–65.87) [16]	*Postop complications:* OR 3.13 (1.65–5.92)–6 [13, 16] RR 1.59 (1.25–2.01)–1.75 (1.28–2.41) [16] *Length of stay* LOS > 2 days OR 4.2 [13]
Fried	30d mortality OR 2.67 (*p* = 0.029) [17]	*Postop complications:* OR 2.54 (1.12–5.77) [13] Major Cx OR 3.13 (1.65–5.92)—4.1 [13] ≥ Clavien 2 Cx OR 4.08 (*p* = 0.006) [17] Mortality or procedural Cx OR 2.2 (*p* = 0.04) [17] *QoL* Mortality or poor QoL at 6 mo OR 2.21 (*p* = 0.03) [17] *Length of stay* LOS intermediately frail OR 1.49 (1.24–1.8) [13]
GFI	GFI ≥ 5 30d mortality ES 0.08 (0.02–0.21) [15]	*Postop complications:* GFI ≥ 5 Postop Cx ES 0.15 (0.06–0.31) [15] GFI ≥ 3 ≥ Clavien 3a OR 3.62 [13] *Length of stay* GFI ≥ 5 ES 7.17 (6.02–8.54) [15] GFI ≥ 3 ES 15.8 (12.79–19.51) [15]
Katz IADL	Dependence in ≥ 1 ADL inpatient mortality OR 1.8 (1.1–3) [23]	
mFI	OR 11–11.7 [13] RR 4.19 (2.96–5.92) [19]	*Postop complications:* OR 11[13] Clavien 4 and 5 postop Cx OR 14.4 [13] mFI > 0.27: Clavien 4 Cx OR 4.8 [13] mFI > 0.12: postop Cx OR 2.71 [13] mFI > 0: postop Cx pooled RR 1.48 (1.35–1.61), major postop Cx pooled RR 1.48 (1.35–1.61) [19] *Discharge to care facility:* RR 2.15 (1.92–2.4) [19]
Slow gait speed 5 m ≥ 6 s	OR 2.63 [13]	Mortality or major morbidity OR 2.63 (1.17–5.9)–3.17 (1.7–2.59) [12]

### American Society of Anesthesiologists Physical Status (ASA-PS)

The ASA tool is a simple ranking of physical health status from 1 to 5 (independent—moribund), which can be completed quickly by a wide range of clinicians [27]. It is broadly applied to all ages and to both emergency and elective populations. An association of high ASA grade with postoperative delirium [18, 22, 26] and postoperative mortality as well as complications [24] was reported in older patients undergoing a range of elective surgery. Conversely, a poor AUROC of 0.64 for the ability of the ASA to predict postoperative mortality following oesophagectomy was described [25].

### Frailty

Of the multitude of frailty tools applied to older surgical patients across nine reviews [12–17, 19, 21, 23], including cardiothoracic surgical patients [12, 13, 17], the modified frailty index (mFI) and Fried criteria were the most frequently reported, followed by the Comprehensive Assessment of Frailty (CAF), Groningen Frailty Index (GFI) and Balducci frailty criteria. A strong association between the mFI and postoperative mortality and Clavien–Dindo grade 4 or 5 postoperative complications were reported in frail patients undergoing mixed major surgery [13]. In a meta-analysis and systematic review, frail patients (defined as any mFI score > 0) had a higher 30-day mortality (RR 4.19, CI 2.96–5.92), higher major postoperative complications (RR 2.03, CI 1.26–3.29) and an higher likelihood of discharge to skilled care accommodation (RR 2.15, CI 1.92–2.4) compared to non-frail patients (mFI score of 0) [19]. Similarly, frail patients meeting at least 3 of 5 phenotypic Fried scale criteria were more likely to die (30-day mortality OR 2.67, *p* = 0.029) [17], develop major postoperative complications [13] and have a longer length of stay (median LOS 9 vs 6 days, *p* = 0.004) [19]. Sandini et al. reported a strong association between frailty and 90-day postoperative mortality [OR 5.77, (CI 4.41–7.55)] and major morbidity [OR 2.56 (CI 2.08–3.16)] in older patients undergoing mixed major surgery, although did not specify a suggested frailty tool [21]. Overall, the majority of frailty tools summarized in this review reported a positive association with morbidity and mortality in older patients undergoing elective surgery.

### Function

Tools to assess function were applied as part of frailty screening and CGA. Gait speed and the timed up and go test (TUGT) were described as bedside preoperative functional tests. Slow gait speed defined as 5 m ≥ 6 s was associated with higher postoperative mortality [13], and composite endpoint of postoperative mortality or major morbidity (OR ranging 2.63 (CI 1.17–5.9) to 3.17 (CI 1.7–2.59) [12, 23]. TUGT over 20 s was associated with postoperative complications [OR ranging from 3.1 (CI 1.1–8.6) to 4.1 (CI 1.6–10.5)] [16] in older patients undergoing oncologic surgery. Clinician or patient-measured functional scales including the Katz, Barthel, Instrumental Activities of Daily Living (IADL) and Nottingham extended ADL scale (NEADL) tools demonstrated an association between functional impairment and increased postoperative mortality [12, 16] and 30-day postoperative complication rate [16].

### Comprehensive geriatric assessment (CGA)

Several objective tools as part of CGA were evaluated and categorized into functional, nutritional, cognitive, mood, comorbidity, polypharmacy and frailty domains [16]. Patients at risk of malnourishment using the Mini Nutritional Assessment (MNA) had a higher risk of short-term postoperative mortality (HR 2.39, CI 1.24–4.61) [16]. Those with a mini-mental status examination (MMSE) score < 24 points had an increased risk of mortality (HR 1.13, CI 1.04–1.22) and postoperative complications (OR 4.55, CI 1.15–18.05) within 6 months following surgery [16]. Older surgical patients with a geriatric depression scale ≥ 5 points were also less likely to survive 6 months (HR 3.62, CI 1.77–7.4) and were more likely to experience postoperative complications [OR range 3.68 (CI 0.96–14.08) to 4.58 (CI 125–16.84)] [16]. Partridge et al. reviewed overall CGA application encompassing the use of objective tools and demonstrated lower postoperative complications and length of stay (4.9 vs 8.9 days, *p* < 0.001) [20].

### Current guidelines on perioperative management of older patients

Recommendations summarized in international guidelines on the perioperative care of older patients are given in Table 3 [28–33]. Most are based on expert consensus opinion. Where validated screening tools have been used to assess individual domains, these are highlighted.

**Table 3:** Current guidelines on perioperative management of older patients

Society	Guideline title	Year	Domain assessed								Evidence
			Cognition	Function	Frailty	Mood	Nutrition	Medication	Comorbidity	Other/comments	
ACS NSQIP/ American Geriatrics Society [28]	Optimal Perioperative Management of the Geriatric Patient	2016	–	–	–	–	–	+ NSQIP 2012	–	References NSQIP 2012 for assessment of individual domains, covers advance directives, preoperative fasting, antibiotic use and venous thromboembolism prevention	Expert opinion
ACS NSQIP/American Geriatrics society [29]	Optimal Preoperative Assessment of the Geriatric Surgical Patient	2012	+ Mini-COG	+ TUGT	+	+ PHQ-2	+	+ AGS Beers Criteria	+ RCRI	Recommends preoperative diagnostic tests including Hb, renal function, serum albumin ± WCC, platelet count, coagulation profile, serum glucose, CXR, ECG, RFT, noninvasive stress testing, BMI and unintentional weight loss	Level 1 + Expert opinion
Association of Anaesthetists Great Britain and Ireland [30]	Perioperative care of the elderly	2014	+ NSQIP 2012	+	+	–	–	+ NSQIP 2012	–	Recommends risk scores such as NSQIP preoperative assessment and Nottingham Hip Fracture Score Recommends multidisciplinary care	Level 1 + Expert opinion
British Geriatric Society [31]	Perioperative Care for Older Patients Undergoing Surgery	2013	+	+	+	–	–	–	+	Social domain assessed	Expert opinion
New South Wales Government Health [32]	The Perioperative toolkit	2018	+	+	+	–	–	+	+	Social domain assessed, involves families in decision-making, multidisciplinary team, patient care pathways, shared decision-making	Expert opinion
Society for Perioperative Assessment and Quality Improvement (SPAQI) [33]	Recommendations for Preoperative Management of Frailty from the SPAQI	2018	+ Mini-COG	+	+ Frailty/Edmonton score	+	–	–	+	Recommends multidisciplinary care and shared decision-making, prehabilitation principles (eg nutritional intervention), requires further studies prior to inclusion in standard recommendations	Level 1 + Expert opinion

*ACS NSQIP* American College of Surgeons National Surgical Quality Improvement Program, *TUGT* timed up and go test, *PHQ-2* Patient Health Questionnaire 2, *RCRI* revised cardiac risk index

The American College of Surgeons National Surgical Quality Improvement Program (NSQIP) 2012 guideline [29] is one of the earliest publications released in this field. It is relatively prescriptive and recommends specific preoperative testing, such as full blood examination and baseline ECG. Validated domain-specific assessment tools are recommended according to expert consensus. The NSQIP 2016 guideline [28] includes sections relating to the immediate perioperative period. It does not discuss screening tools, however, refer to the NSQIP 2012 guideline where screening tools are discussed in further detail, for example, in the medication management domain [28, 29].

The guidelines of the Association of Anaesthetists of Great Britain and Ireland [30] similarly refer to NSQIP 2012 for assessment of domains including cognition and medication management. These guidelines also recommend preoperative risk score calculation tailored to specific surgical situations, for example, use of the Nottingham Hip Fracture Score in the prediction of 30-day mortality after hip fracture surgery [30]. The British Geriatric Society guideline [31] and an Australian guideline, the New South Wales Government Health Perioperative toolkit [32], recommend assessing several domains to risk stratify patients, but do not specify which tools to use. Both these guideline emphasize the importance of assessing social domains which are not included in NSQIP guidelines [31, 32]. The Society for Perioperative Assessment and Quality Improvement (SPAQI) [33] covers several domains including cognition, functional status, frailty, mood disorder and medical comorbidity. Specific screening tools are suggested for some of the domains, such as mini-COG to assess cognition. The more recently published guidelines, including SPAQI and the New South Wales Government Health Perioperative Toolkit, tend to state broader expert consensus recommendations such as multidisciplinary care and shared decision-making [32, 33].

Overall, there is heterogeneity in the approach taken by each guideline committee towards the perioperative management of older patients. Assessment domains and tools differ between guidelines. Almost all guidelines recommend an assessment of cognition, functional status and frailty, although many do not specify which tool to use.

## Discussion

This scoping review of systematic reviews and meta-analyses demonstrates the broad range of tools that are applied preoperatively to older patients undergoing elective surgery. The most commonly described tools include the ASA, frailty tools and tools utilized during CGA. The majority of tools show a positive association with short-term postoperative mortality and morbidity as measures of postoperative recovery in various older surgical patient populations, including cardiothoracic patients. Due to the differences in utilized cut-off points and outcome parameters, tools are unable to be compared in order to support one tool over another. Perioperative guidelines offer recommendations for pre-assessment approach in older surgical patients but lack consensus regarding the selection of preoperative tools. As a result, there is no evidence to support a distinct tool which should be applied universally to older surgical patients.

The ASA is simple to apply and routinely used by anaesthetists to broadly stratify patients in all perioperative settings. Whilst there is a consistent association between a higher ASA score and poor postoperative outcomes [34], it remains a subjective score with high inter-observer variability [35].

The inherent value of identifying frailty, defined as an age-related cumulative decline in multiple physiological systems [36], has been increasingly recognized as a measure of high-risk in older surgical patients [9, 37, 38]. However, standardized assessment is often lacking due to the absence of a universal or ‘gold standard’ frailty tool as demonstrated in this review.

CGA is a time-consuming patient-specific evaluation which might not be appropriate to administer to all older patients preoperatively [39]. Whilst there is supportive evidence for CGA in both emergency [40] and elective [41] older surgical patients, it requires specialist training to administer the domain-specific tools [20, 21]. Adaptations of the CGA into screening tools such as the G-8 questionnaire [42] and CGA-GOLD [43] require further research in broad surgical populations and were not published in a meta-analysis or systematic review format for inclusion. Additional commonly utilized screening tools did not meet the inclusion criteria for this review. For example, the P-POSSUM uses intraoperative variables [44] and the Revised Cardiac Risk Index was only included in one systematic review within our literature search [45].

International guidelines are fairly consistent in terms of recommending a complete preoperative medical assessment based on geriatric domains included in a CGA. Most recommendations are based on expert opinion. Although cognition, functional status and frailty are consistently prioritized, with corresponding tools given as an example in each guideline, there is no consensus regarding which tool to use. This suggests that completing any chosen assessment may be more important than which tools are specifically used. The ease of use of the guidelines and ability to apply the recommendations quickly and effectively in an outpatient setting, such as a preadmission clinic, has not been validated. Furthermore, a comprehensive approach might not necessary for all older patients.

The 2018 Royal College of Surgeons High-Risk General Surgical Guideline recommends all patients undergo risk assessment prior to surgery and classifies patients with a predicted postoperative mortality risk of ≥ 5% as high risk [9]. This can be estimated using a preoperative risk assessment tools and frailty assessment. Resources can consequently be targeted towards high-risk patients including planning postoperative critical care beds, senior anaesthetic and surgical intraoperative presence and engagement of the multidisciplinary perioperative team. Whilst no screening tool has been identified as the single best option in the older general surgical patient, it appears that making a screening assessment using any validated tool to guide the application of comprehensive geriatric assessment is warranted. Given the shared recommendation of guidelines to assess cognition, functional status and frailty, it is reasonable for clinicians to choose a tool within one or all of these domains.

There were limitations met throughout this scoping review which contributed to the inability to define a single appropriate screening tool. The high number of tools reported and marked heterogeneity in outcomes measured significantly limited the ability to compare tools in this review. Whilst narrowing the search to a more specific population may have been more achievable, we aimed to find a broadly applicable tool to reflect clinical need and simplify perioperative pathways. There were multiple selection biases including skewed subsurgical groups, the underrepresentation of oldest old patients and geography.

Geriatrician-led multidisciplinary perioperative care targeting older patients undergoing surgery is growing in clinical practice. The establishment of the ‘Perioperative Care of Older Patients Undergoing Surgery’ (POPS) service in the UK is an example of a successful collaborative perioperative model for older patients, which has led to improved mortality and morbidity in older surgical patients [41, 46]. In this model, preoperative screening is not limited to a specific tool but encourages identification of geriatric syndromes and clinical judgement [47]. Despite strong evidence and UK national endorsement of the POPS model of care, clinical uptake is not yet widely disseminated with an acknowledged ‘implementation gap’. A logic implementation model of the POPS service has successfully led to translation of core components to a smaller setting [48].

## Conclusion

The use of screening tools to predict postoperative outcomes in older patients prior to elective surgery is important in identifying high-risk patients and developing safe, efficient and effective clinical pathways for the perioperative team. A number of screening tools have been identified as associated with poor postoperative outcomes and the selection of a frailty, functional and/or cognitive tool is proposed. International consensus guidelines recommend a complete and thorough medical and geriatric assessment of the older patient prior to surgery; screening tools can help guide which patients will benefit from this comprehensive approach.

## Author contributions and authorship

All authors meet the International Committee of Medical Journal Editors (ICMJE) criteria for authorship. RA: 40% contribution (study design, literature search, results Tables 1, 2, Fig. 1, manuscript drafting and editing, reference collation). NSH: 40% contribution (study design, literature search, review of guidelines, results Table 3, manuscript drafting and editing). ABM: 20% contribution (study design, manuscript editing, research supervisor).

## Electronic supplementary material

Below is the link to the electronic supplementary material.

Supplementary file1 (DOCX 13 kb)
