# Supplementary material for: Which preoperative screening tool should be applied to older patients undergoing elective surgery to predict short-term postoperative outcomes? Lessons from systematic reviews, meta-analyses and guidelines
Source: Intern Emerg Med. 2020 Jul 1;16(1):37–48. doi: 10.1007/s11739-020-02415-y (PMC7843484; doi:10.1007/s11739-020-02415-y)
Supplement: Supplementary file 1 — Supplementary file1 (DOCX 13 kb) [file 11739_2020_2415_MOESM1_ESM.docx]

**Appendix 1 – MEDLINE Ovid Search String**

1 Surgical Procedures, Operative/ (52629)

2 surger*.mp. [mp=title, abstract, original title, name of substance word, subject heading word, keyword heading word, protocol supplementary concept word, rare disease supplementary concept word, unique identifier, synonyms] (1136662)

3 Elective Surgical Procedures/ (11418)

4 (elective adj3 (surgery or Surgical)).mp. [mp=title, abstract, original title, name of substance word, subject heading word, keyword heading word, protocol supplementary concept word, rare disease supplementary concept word, unique identifier, synonyms] (27232)

5 1 or 2 or 3 or 4 (1160157)

6 screening tool*.mp. [mp=title, abstract, original title, name of substance word, subject heading word, keyword heading word, protocol supplementary concept word, rare disease supplementary concept word, unique identifier, synonyms] (17265)

7 Risk Assessment/ or Risk Factors/ (867473)

8 "Outcome Assessment (Health Care)"/ (62221)

9 Decision Support Techniques/ (16997)

10 ((pre-operative or pre-surg*) adj3 (screen* or evaluat* or assessment*)).mp. [mp=title, abstract, original title, name of substance word, subject heading word, keyword heading word, protocol supplementary concept word, rare disease supplementary concept word, unique identifier, synonyms] (2908)

11 (risk adj3 (assess* or stratification* or quantif*)).mp. [mp=title, abstract, original title, name of substance word, subject heading word, keyword heading word, protocol supplementary concept word, rare disease supplementary concept word, unique identifier, synonyms] (303550)

12 6 or 7 or 8 or 9 or 10 or 11 (1013860)

13 outcome*.mp. [mp=title, abstract, original title, name of substance word, subject heading word, keyword heading word, protocol supplementary concept word, rare disease supplementary concept word, unique identifier, synonyms] (2003455)

14 Postoperative Complications/ (327836)

15 Mortality/ or Morbidity/ (63255)

16 Length of Stay/ (74702)

17 Treatment Outcome/ (823314)

18 (Mortality or morbiditi* or complication* or outcome* or length of stay or "LOS").mp. [mp=title, abstract, original title, name of substance word, subject heading word, keyword heading word, protocol supplementary concept word, rare disease supplementary concept word, unique identifier, synonyms] (3375851)

19 13 or 14 or 15 or 16 or 17 or 18 (3388758)

20 (tool* or indic* or index or measure* or scale* or algorithm* or score* or model*).mp. [mp=title, abstract, original title, name of substance word, subject heading word, keyword heading word, protocol supplementary concept word, rare disease supplementary concept word, unique identifier, synonyms] (8931620)

21 (frail* or frailty*).mp. [mp=title, abstract, original title, name of substance word, subject heading word, keyword heading word, protocol supplementary concept word, rare disease supplementary concept word, unique identifier, synonyms] (19352)

22 20 or 21 (8940041)

23 5 and 12 and 19 and 22 (38238)

24 limit 23 to (meta analysis or systematic reviews) (2837)
